# *Cysticercus pisiformis*-derived novel-miR1 targets TLR2 to inhibit the immune response in rabbits

**DOI:** 10.3389/fimmu.2023.1201455

**Published:** 2023-07-25

**Authors:** Guoliang Chen, Guiting Pu, Liqun Wang, Yanping Li, Tingli Liu, Hong Li, Shaohua Zhang, Xuelin Wang, Xiaolei Liu, Xuenong Luo

**Affiliations:** ^1^ State Key Laboratory for Animal Disease Control and Prevention, Key Laboratory of Veterinary Parasitology of Gansu Province, Lanzhou Veterinary Research Institute, Chinese Academy of Agricultural Sciences, Lanzhou, Gansu, China; ^2^ Key Laboratory for Zoonosis Research of the Ministry of Education, Institute of Zoonosis, and College of Veterinary Medicine, Jilin University, Changchun, China; ^3^ Jiangsu Co-Innovation Center for the Prevention and Control of Important Animal Infectious Disease and Zoonoses, Yangzhou University, Yangzhou, China

**Keywords:** rabbit, Cysticercosis pisiformis, novel-miR1, TLR2, NFκB

## Abstract

Cysticercosis pisiformis, a highly prevalent parasitic disease worldwide, causes significant economic losses in the rabbit breeding industry. Previous investigations have identified a novel microRNA, designated as novel-miR1, within the serum of rabbit infected with *Cysticercus pisiformis*. In the present study, we found that *C. pisiformis*-derived novel-miR1 was released into the rabbit serum via exosomes. Through computational analysis using TargetScan, miRanda, and PITA, a total of 634 target genes of novel-miR1 were predicted. To elucidate the functional role of novel-miR1, a dual-luciferase reporter assay was utilized and demonstrated that novel-miR1 targets rabbit Toll-like receptor 2 (TLR2). Rabbit peripheral blood lymphocytes (PBLCs) were transfected with novel-miR1 mimic and mimic NC, and the *in vitro* experiments confirmed that novel-miR1 suppressed the expression of pro-inflammatory cytokines such as TNF-α, IL-1β, and IL-6 through the nuclear factor kappa B (NF-κB) pathway. *In vivo* experiments demonstrated that novel-miR1 was significantly upregulated during the 1–3 months following infection with *C. pisiformis in* rabbits. Notably, this upregulation coincided with a downregulation of TLR2, P65, pP65, TNF-α, IL-1β, and IL-6 in PBLCs. Collectively, these results indicate that the novel-miR1 derived from *C. pisiformis* inhibited the rabbits’ immune response by suppressing the NF-κB-mediated immune response. This immune modulation facilitates parasite invasion, survival, and establishment of a persistent infection.

## 1 Introduction

*Cysticercus pisiformis* is a widely prevalent larval form of tapeworm that primarily parasitizes the liver, omentum, and mesenteries of lagomorphs and rodents, leading to the development of cysticercosis pisiformis (1, 2). Among these hosts, rabbits are the most frequently affected and suffer significant consequences from cysticercosis pisiformis. This parasitic infection adversely affects rabbit reproductive rates and poses a serious threat to endangered rabbit species such as the teporingo (*Romerolagus diazi*) (3, 4). Moreover, it leads to decreased body weight and cholesterol levels, even leading to death (5). Importantly, *C. pisiformis* infection in rabbits impairs their immune responses, rendering them susceptible to secondary infections by other pathogens (6), which has substantial economic impacts in the rabbit breeding industry and impacts animal welfare.

Exosomes, extracellular vesicles ranging in size from 40 to 160 nm, are released by various cells, including both prokaryotes and eukaryotes (7). Numerous studies have reported that exosomes are mediators of vesicle transport, playing a pivotal role in parasite–host interactions (8–11). *Trichinella spiralis* excretory/secretory products have been shown to modulate programmed death 1-mediated M2 macrophage polarization (12). Exosomes derived from *T. spiralis* impact the nuclear factor kappa B (NF-κB) and mitogen-activated protein kinase (MAPK) signaling pathways, thereby inhibiting M1 macrophage polarization (13). Similarly, *Taenia asiatica* exosomes inhibit LoVo cell proliferation and autophagy via the Adenosine 5'-monophosphate (AMP)-activated protein kinase (AMPK) pathway (14). Notably, miRNAs are essential cargo within exosomes and are the key functional units (15, 16). *Echinococcus multilocularis* exosomes containing emu-miR-4989 induces UBE2N suppression (17). *Fasciola hepatica* releases fhe-miR-125b through exosomes to target the mammalian Argonaut protein (Ago-2) within macrophages during infection to downregulate the production of inflammatory cytokines (18). miR-1-3p and let-7-5p expressed by *T. spiralis* larvae-derived extracellular vesicles promote the polarization of bone marrow macrophages toward the M2b type while inhibiting fibroblast activation (19).

MicroRNAs (miRNAs) are small noncoding RNAs that play important roles in posttranscriptional gene silencing (20). They are capable of binding to the 3′-untranslated region (UTR), coding region, or 5′-UTR of the target mRNA to inhibit translation or facilitate mRNA degradation in a wide variety of physiological and pathological processes, including growth, proliferation, differentiation, development, metabolism, infection, immunity, and cell death (21, 22). Data from numerous studies have shown that miRNA expression profiles are altered during parasite infection and suggest that differentially expressed miRNAs play important roles in host immune responses (18, 23–25).

Toll-like receptors (TLRs) are pattern recognition receptors (PRRs) that play a crucial role in recognizing pathogen-associated molecular patterns (PAMPs) and activate intracellular signaling pathways, leading to the induction of inflammatory cytokine genes such as TNF-α, IL-6, IL-1β, and IL-12 (26, 27). Among the TLRs, TLR2 is involved in the recognition of various PAMPs derived from bacteria, fungi, parasites, and viruses (28). Upon recognition of PAMPs, including proteins, lipoproteins, lipids, nucleic acids, and endogenous ligands, TLR2 induces interferon (TIRF), leading to the activation of MAPKs through the MyD88 pathway or the Toll/IL-1R-related adaptor protein, resulting in the translocation of NF-κB and subsequent transcription and synthesis of pro-inflammatory cytokines (29).

Helminths, a class of pathogens, also elicit immune responses through TLR2. For instance, *Clonorchis sinensis* heat shock cognate B (CsHscB) activates TLR2 to induce the expression of IL-10, exerting immune regulatory activities (30). During *T. spiralis* infection, TLR2 is upregulated in the mouse small intestine, suggesting its crucial role in host defense mechanisms against this parasite (31). TLR2 is also important for the development of a Th1-dominant immune response in the mouse model of *Taenia crassiceps* cysticercosis (32). Cystic echinococcosis-derived antigens, including hydatid cyst fluid (HCF), germinal layer antigens (GL), somatic and excretory/secretory (ES) products of protoscoleces (PSC), have been shown to downregulate the expression of TLR2 in ovine peripheral blood leukocytes, potentially contributing to the establishment of chronic infection by suppressing host immunity (33).

Therefore, TLR2 plays important roles in the induction of innate immune responses. Despite the knowledge on the important roles played by parasite-derived miRNAs during infection, little is known about the role of TLR2 as a pattern recognition receptor in the context of *C. pisiformis* infection in rabbits. In our experiments, we observed a significant upregulation of novel-miR1, derived from *C. pisiformis*, in the serum of infected rabbits. Furthermore, TLR2 was predicted and verified as a target of novel-miR1. This study aims to investigate the mechanism underlying the interaction between novel-miR1 and TLR2 in rabbits infected with *C. pisiformis*. The findings will contribute valuable insights into the mechanism of *C. pisiformis* infection, thereby benefiting the diagnosis and treatment of cysticercosis pisiformis and providing strategies for the prevention and control of helminth infections.

## 2 Methods

### 2.1 Rabbit and *C. pisiformis* collection

Fifty-day-old New Zealand White rabbits weighing 1.5~2 kg were obtained from the Laboratory Animal Center of Lanzhou Veterinary Research (Lanzhou, China). The rabbits were allowed to adaptively acclimate for 1 week prior to the experiment. A total of six rabbits were orally infected with 1 ml of infective *Taenia pisiformis* eggs (1,000 eggs/ml), establishing the *C. pisiformis* infection group (CPi). As a negative control group (NC), three rabbits were administered 1 ml of phosphate-buffered saline (PBS) via oral gavage. All rabbits were reared with the same forage and clean water. Following the collection of required blood samples, necropsy was performed on the six rabbits to confirm *C. pisiformis* infection. The examination results are shown in **Supplementary Table S1**.

All rabbit experiments conducted in this study were approved by the Animal Ethics Committee of Lanzhou Veterinary Research Institute, Chinese Academy of Agricultural Sciences (Permit No. LVRIAEC-2016-006), and the protocols used were strictly in accordance with the guidelines of animal welfare. Every effort was made to minimize any potential harm to the experimental rabbits in accordance with the study protocols.

### 2.2 Cells and media

Rabbit peripheral blood lymphocytes (PBLCs) were isolated from 5-ml fresh rabbit blood samples collected in tubes containing EDTA and separated using rabbit peripheral blood lymphocytes isolation kit based on Ficoll density gradient centrifugation (TBD Science, China, LTS10965). Cell viability was tested by trypan blue staining (TBD Science, China, TBD20180079) following the manufacturer’s instructions. The isolated PBLCs were cultured in RPMI 1640 medium (Gibco, Grand Island, NY, USA) supplemented with 10% fetal bovine serum (FBS) (Gibco), penicillin (100 unit/ml) (Gibco), and streptomycin (100 µg/ml) (Gibco).

HEK293T cells were cultured in DMEM (Gibco) containing 10% FBS, penicillin (100 unit/ml), and streptomycin (100 µg/ml). All cells were cultured at 37°C in a 5% CO_2_ cell culture incubator.

### 2.3 Exosome isolation and identification

*T. pisiformis* larvae were harvested from the peritoneal cavities of CPi rabbits and cultured in 10 ml RPMI 1640 medium (Gibco, Grand Island, NY, USA) supplemented with 10% exosome-depleted FBS and antibiotics (100 U/ml penicillin, 100 μg/ml streptomycin) at 37°C in 5% CO_2_ cell culture incubator. After 12 h, the culture media were collected and replaced with fresh medium. The collected media were centrifuged at 2,000×g at 4°C for 10 min to remove cell debris, and the exosomes were isolated using the Minute™ Hi-Efficiency Exosome Isolation Reagent (Cat. No. EI-027; Invent Biotechnologies, Inc., Plymouth, MN, USA) following the manufacturer’s protocol. The exosome precipitate was resuspended in 500 μl phosphate-buffered saline (PBS) filtered through a 0.2-μM pore filter. The concentration of exosomes was determined using a Pierce BCA Protein Assay Kit (Thermo Fisher Scientific, Waltham, MA, USA). The particle size of the exosomes was analyzed using a NanoSight LM10 instrument (Nanosight, Wiltshire, UK). The exosome size and morphology were observed through transmission electron microscopy (TEM, Hitachi Ltd., Tokyo, Japan). Briefly, exosomes were adsorbed onto a 200-mesh formvar-coated copper grid (Agar Scientific Ltd., Stansted, UK) and incubated for 10 min at room temperature. After staining with 2% tungstophosphoric acid solution for 1 min, the exosomes were examined under an electron microscope (34).

### 2.4 Exosome labeling and confocal laser scanning microscope analysis

To visualize exosomal uptake by rabbit PBLCs, we labeled exosomes with DiD dye (ab275319, Abcam, Cambridge, MA, USA). A total of 6 µg of exosomes were mixed with 2 μl DiD dye (0.2%) and diluted to 1 ml PBS (35). As a negative control, only DiD dye mixed with PBS was used. The mixture was then incubated at 37°C for 30 min to allow for labeling of the exosomes. The labeled exosomes were subsequently washed with PBS and centrifuged at 120,000×g and 4°C for 1 h. The resulting precipitate was resuspended in 180 μl of PBS.

Subsequently, the labeled exosomes were added to three wells of a 12-well plate containing rabbit PBLCs and incubated for 12 h in a 5% CO_2_ incubator at 37°C. Following incubation, the cells were stained with 10 µg/ml of 4,6 diamidino-2-phenylindole (DAPI) (Beyotime, Shanghai, China) for 5 min. The stained PBLCs were added to a glass slide and covered with a coverslip for imaging under a confocal laser scanning microscope (Nikon, Tokyo, Japan) (36).

### 2.5 Novel-miR1/siRNA transfection and exosome treatment

PBLCs were isolated from fresh peripheral blood from healthy rabbits. The isolated cells were diluted to 2~10 × 10^6^ cells/ml in RPMI 1640 medium supplemented with 10% FBS, penicillin (100 unit/ml), and streptomycin (100 µg/ml). Subsequently, 1 ml of the PBLC suspension was added to each well of a 12-well plate and cultured in a 37°C incubator with 5% CO_2_. For transfection experiments, 10 pmol/L of novel-miR1 mimic, mimic NC, inhibitor, inhibitor NC, siTLR2, and siRNA NC (RiboBio, Guangzhou, China) were transfected into the PBLCs using 2 μl of Lipofectamine RNAiMAX transfection reagent (Thermo Fisher Scientific, USA) for a duration of 24 h. In exosome treatment experiments, 20 µg of total exosome protein or an equivalent volume of PBS was added per well and coincubated for 24 h.

### 2.6 Extraction of miRNAs and RNA

The miRNAs were separated from fresh blood samples or *C. pisiformis*-derived exosomes using an EasyPure miRNA Kit (TransGen, China) according to the manufacturer’s instructions. Total RNA was isolated from the PBLCs and *C. pisiformis* using TRIzol reagent (Thermo Fisher Scientific) following the manufacturer’s protocol.

### 2.7 Real-time qPCR analysis

The expression of novel-miR1 and TLR2 in serum miRNAs or PBLC RNA in CPi and NC groups was analyzed using real-time quantitative PCR (qPCR). The miRNAs were reverse-transcribed into cDNA using the Mir-XTM miRNA First-Strand Synthesis Kit (Clontech, Mountain View, CA, USA). The cDNA first-strand synthesis of PBLC total RNA was performed using the HiScript^®^ III 1st Strand cDNA Synthesis Kit (Vazyme, China). All cDNAs were used for real-time fluorescence qPCR detection using an ABI 7500 quantitative PCR instrument (Thermo Fisher Scientific). qPCR was performed in a 20-μl reaction volume, including 10 μl SYBR Advantage Premix (2×) (Takara, Japan), 2 μl cDNA, 0.4 μl ROX Dye (50×), 0.8 μl specific forward primer, 0.8 μl specific reversed primer, and 6 μl RNase-free water. qPCR was performed with the following: 95°C for 10 min and 40 cycles of 95°C for 15 s and 60°C for 34 s. The relative expressions were normalized to U6 small nuclear RNA (snRNA) or GAPDH. All primers were designed and produced by Sangon Biotech (Sangon Biotech, China) (**Table 1**). The relative expression of novel-miR1 was calculated using the 2^−ΔΔCt^ method (37).

**Table 1:** Primer sequences used in quantitative PCR.

Primer name	Primer sequence (5′–3′)
Novel-miR1	TATACGCAGGTGCGAAAGCAGG
IL-17RB-F	ACGAGACGACAGTCCAAGTG
IL-17RB-R	GTGGAAAACCCAATCACGGC
CEBPA-F	TTCAGGAGTAACCGTGTGCC
CEBPA-R	CGGCAGAAACCCTCCAAGTA
CASP9-F	CTGTTTCCGAGCGAGGGATT
CASP9-R	CCTGGCCCCGCTAAGTTTTA
TLR2-F	CGCTGAAAAACCTGACCGAC
TLR2-R	TGTGTATCCGTGTGCTGGAC
MAPK12-F	CCTACTTCGAGTCCCTGCAC
MAPK12-R	AAGTGACACGCTTCCACTCG
GSAP-F	GACCTCATGTGCCGCATACT
GSAP-R	ACTGCCACGCCTATTGAAGT
IL10RA-F	TCCCCGCTTGCAATTCTCAT
IL10RA-R	TGTGAGCTGACCACACACTG
IRF8-F	AGGCTCGCGGGTTTATGATT
IRF8-R	CTTCGCCGTCGCACATTAAG
TNF-α-F	GACGGGCTGTACCTCATCTAC
TNF-α-R	GACCTTGTTCGGGTAGGAGAC
IL-1β-F	GGCAGGTCTTGTCAGTCGTT
IL-1β-R	AGTTCTCAGGCCGTCATCCT
IL-6-F	AGAACCATCGAGAGCATCCG
IL-6-R	GCCTTGGAAGGTGCAGATTG
IL-10-F	GTCACCGATTTCTCCCCTGT
IL-10-R	GATGTCAAACTCACTCATGGCT
GAPDH-F	TTGAAGGGCGGAGCCAAAA
GAPDH-R	CAGGATGCGTTGCTGACAATC

To demonstrate the source of novel-miR1 in rabbit serum, an absolute quantification qPCR was performed. Total RNA (1 μg) from all samples, including rabbit serum (CPi and NC), *C. pisiformis*, and *C. pisiformis* exosomes, was reverse-transcribed into cDNA using the Mir-XTM miRNA First-Strand Synthesis Kit. qPCR was conducted on an ABI 7500 using a TransStart Tip Green qPCR SuperMix Kit (TransGen Co., Beijing, China) with the following protocol: initial denaturation at 95°C for 30 s, followed by 40 cycles of 95°C for 15 s and 60°C for 34 s. The copy number of novel-miR1 in the samples was determined using a standard curve established with 10-fold serial dilutions (1 × 10^7^ to 1 × 10^2^ copies/μl) of the standard novel-miR1.

### 2.8 Function prediction of novel-miR1

To investigate the potential functional roles of novel-miR1, we employed bioinformatics tools to predict its target genes. The software TargetScan (http://www.targetscan.org/), miRanda (http://www.microrna.org/), and PITA (http://www.pita.org.fj/) were utilized, and the analysis was conducted on the *Oryctolagus cuniculus* genome (https://www.ncbi.nlm.nih.gov/genome/316?genome_assembly_id=1549366). The predicted miRNA target genes were determined by selecting the intersection set of results obtained from the three software programs. The functions of the identified target genes were analyzed by Gene Ontology (GO) functional annotation clustering analysis and Kyoto Encyclopedia of Genes and Genomes (KEGG) pathway analysis. These analyses were performed using the online DAVID bioinformatics database functional annotation tool (https://david.ncifcrf.gov/).

### 2.9 Luciferase reporter assay

HEK293T cell lines were employed for the co-transfection experiments. The novel-miR1 mimic or mimic NC was cotransfected with dual-luciferase reporter plasmids (pmir-GLO-TLR2, pmir-GLO-TLR2-WT, or pmir-GLO-TLR2-Mut). The luciferase reporter assay was performed using the Dual-Luciferase Reporter Assay System (Promega, Madison, WI, USA). For accurate comparisons, firefly luciferase activity was normalized to the Renilla luciferase activity. The effect of a novel-miR1 on the luciferase reporter constructs containing the TLR2 3′-UTR or the corresponding mutant was determined by comparing the reporter activity with the control. Each luciferase reporter assay was conducted in triplicate.

PBLCs proteins were extracted using RIPA lysis buffer supplemented with 1% protease inhibitor. Here, 30 µg total proteins were incubated at 100°C for 10 min. The protein samples were separated on a 10% SDS-PAGE gel alongside prestained protein markers. The separated proteins were transferred to a PVDF membrane and blocked with 5% skimmed milk at room temperature for 3 h. Rabbit anti-TLR2 monoclonal antibody (1:1,000; Abcam; cat. no. Ab209217), phospho-NF-κB p65 (Ser536) rabbit monoclonal antibody (1:1,000; Abcam; cat. no. ab239882), NF-κB p65 rabbit polyclonal antibody (1:1,000; Abcam; cat. no. Ab16502), rabbit anti-IL-6 polyclonal antibody (1:1,000; Bioss, Woburn, MA, USA; cat. no. bs-0782R), rabbit anti-TNF-α polyclonal antibody (1:1,000; Bioss; cat. no. bs-2081R), mouse anti-IL-1β monoclonal antibody (1:500; Santa Cruz Biotechnology, Dallas, TX, USA; cat. no. sc52012), and mouse anti-β-actin monoclonal antibody (1:2,000 dilution, Beyotime Biotechnology, China) were used as the primary antibodies in this study. The secondary antibodies used were goat anti-mouse IgG/HRP antibody (1:4,000 dilution, Beyotime Biotechnology) or goat anti-rabbit IgG/HRP antibody (1:4,000 dilution, Beyotime Biotechnology), as per the manufacturer’s instructions. The membrane was reacted with ultrasensitive ECL luminescence reagent (Beyotime Biotechnology) for color detection.

### 2.10 Cytokine analysis

Enzyme-linked immunosorbent assay (ELISA) was performed to quantify the levels of IL-6 (ml027844), TNF-α (ml028087), and IL-1β (ml027165). ELISA kits were obtained from Shanghai Enzyme-linked Biotechnology Co. Ltd. (Shanghai, China). PBLCs were isolated from the blood of CPi and NC rabbits and the cell culture supernatants were collected to measure the cytokine levels following the manufacturers’ instructions.

### 2.11 Statistical analysis

The Student’s t-test method was used to analyze the significance of the miRNA expression, ImageJ was used for Western blot grayscale analysis, and GraphPad 9 was used to create the relative expression diagram. p < 0.05 was considered statistically significant.

## 3 Results

### 3.1 Novel-miR1 derived from *C. pisiformis* exosomes was identified in infected rabbit serum

**Figure 1:**
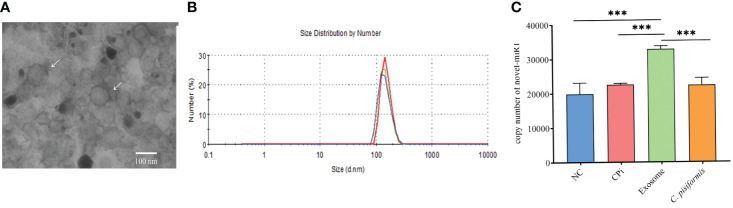
Identification and analysis of novel-miR1 source and target genes. **(A)** Morphological characterization of *C. pisiformis* exosomes through transmission electron microscope (TEM). **(B)** Size distribution of exosome of *C. pisiformis* by nanoparticle tracking analysis (NTA). **(C)** Quantification of novel-miR1 copy numbers in different samples. NC represents the negative control rabbit serum miRNA, CPi represents the *C. pisiformis*-infected rabbit serum total miRNA, exosomes are the *C. pisiformis* exosome total miRNA, and *C. pisiformis* represents the *C. pisiformis* total miRNA. Novel-miR1 copy numbers were determined by absolute quantitative PCR using 20 ng total miRNAs. *** indicates a significance level of p ≤ 0.001.

Exosomes derived from *C. pisiformis* were successfully isolated, and their measured size was about 100 nm (**Figures 1A, B**). To better understand the origin of the novel-miR1 in rabbit serum, absolute qPCR was used to detect the levels of miRNA in samples obtained from rabbit blood (CPi, NC), *C. pisiformis*, and *C. pisiformis* exosomes. The results revealed a significant enrichment of novel-miR1 within the *C. pisiformis* exosomes (**Figure 1C**). These findings indicate that novel-miR1 in infected rabbit serum is released into the bloodstream through *C. pisiformis* exosomes.

### 3.2 TLR2 is targeted by novel-miR1

**Figure 2:**
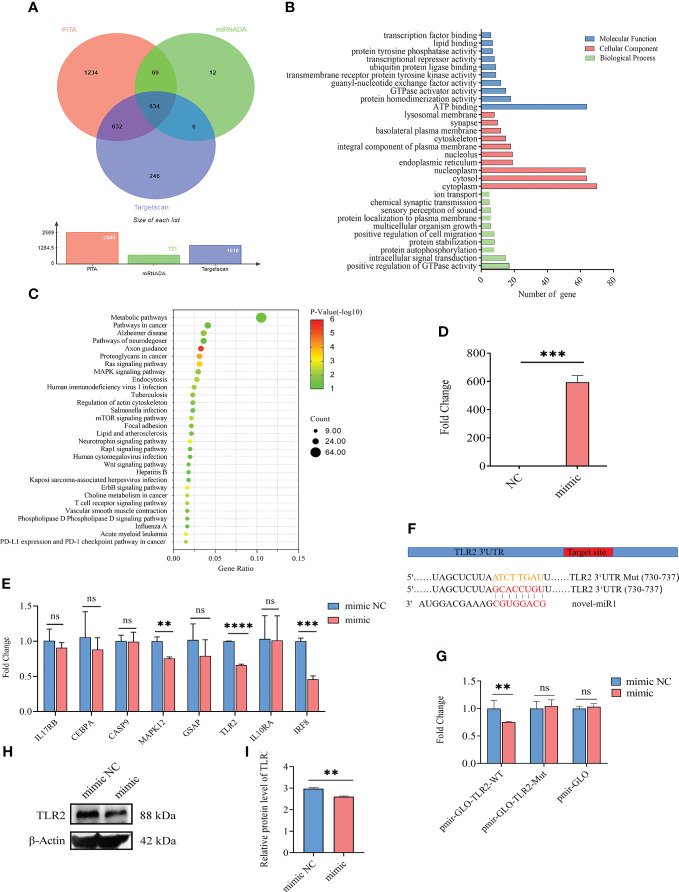
Targeting of *TLR2* 3′-UTR by novel-miR1. **(A)** Venn diagram showing the intersection of predicted target genes from different software programs, namely, PITA, miRanda, and TargetScan. **(B)** Gene Ontology (GO) molecular function annotations of the target genes of novel-miR1. The top 10 GO terms for biological processes, cellular component, and molecular function are shown based on their respective p-values. **(C)** Kyoto Encyclopedia of Genes and Genomes (KEGG) pathway analysis of the predicted target genes of novel-miR1. The size of dots indicates the number of genes associated with each pathway. **(D)** Overexpression of novel-miR1 in rabbit PBLCs. **(E)** Relative expression of novel-miR1 target genes in rabbit PBLCs after novel-miR1 treatment. **(F)** novel-miR1 binding *TLR2* 3′-UTR site and the sequences of luciferase reporter plasmids (pmir-GLO-TLR2-WT and pmir-GLO-TLR2-Mut). **(G)** Luciferase reporter assay results indicating the downregulation of *TLR2* through binding to its target 3′-UTR. **(H)** Western blot assay results showing the downregulation of TLR2 protein in PBLCs by novel-miR1. **(I)** Western blot assay results demonstrating the downregulation of TLR2 protein in PBLCs by novel-miR1 (fold change was calculated by the gray values of the image). ns, p ≥ 0.05; **, p ≤ 0.01; ***, p ≤ 0.001; ****, p ≤ 0.0001.

A total of 634 target genes were predicted for novel-miR1 using TargetScan, miRNADA, and PITA (**Figure 2A**, **Supplementary Table S2**). GO functional annotation clustering analysis (**Supplementary Table S3**) revealed enrichment of novel-miR1 target genes in various biological processes (BPs), such as positive regulation of GTPase activity, intracellular signal transduction, protein autophosphorylation, protein stabilization, and ion transport. Molecular function (MF) analysis highlighted ATP binding, protein homodimerization activity, guanyl-nucleotide exchange factor activity, and transcription factor binding as enriched functions. Cellular component (CC) analysis showed localization to cytoplasm, nucleoplasm, integral component of plasma membrane, and lysosomal membrane (**Supplementary Table S3**). The top 10 terms for BP, MF, and CC are presented in **Figure 2B**. KEGG pathway analysis revealed the involvement of novel-miR1 target genes in 60 signaling pathways. Notably, these pathways included crucial signaling cascades such as Ras, MAPK, mTOR, Wnt, and ErbB. Moreover, the target genes were found to cluster in pathways related to pathogenic infection and immune responses, such as pathways in cancer, human immunodeficiency virus 1 infection, tuberculosis, *Salmonella* infection, human cytomegalovirus infection, hepatitis B, Kaposi sarcoma-associated herpesvirus infection, Influenza A, and T-cell receptor (**Supplementary Table S4**). **Figure 2C** illustrates the top 30 signaling pathways represented by the novel-miR1 target genes, highlighting the significant role of novel-miR1 in pathogenic infection.

To further investigate the immunoregulatory function of novel-miR1 in rabbit serum, rabbit PBLCs were transfected with novel-miR1 mimic to achieve overexpression of novel-miR1 (850-fold change, **Figure 2D**). Eight immune-related target genes (*IL17RB, CEBPA, CASP9, MAPK12, GSAP, TLR2, IL10RA, *and *IRF8*) were selected for validation by qPCR analysis. Compared to the control group, novel-miR1 overexpression significantly inhibited the expression of *MAPK12, TLR2*, and *IRF8* (p < 0.05), while *IL17RB, CEBPA,* and *GSAP* exhibited a tendency toward downregulation (**Figure 2E**).

Next, we verified the relationship between novel-miR1 and TLR2 and found that the seed sequence of novel-miR1 can bind to the 3′UTR of *TLR2* (Gene ID: 100009578) using TargetScan software (**Figure 2F**). To verify the targeting relationship between novel-miR1 and TLR2, we conducted a dual luciferase reporter experiment by cotransfecting the vectors pmirGLO-TLR2-WT, pmirGLO-TLR2-Mut, and pmirGLO with novel-miR1 mimic/mimic NC into HEK293T cells. The results showed a decrease in firefly luciferase activity when pmirGLO-TLR2-WT was cotransfected with novel-miR1 mimics. However, differences were not found in the cells cotransfected with novel-miR1 mimics and pmirGLO-TLR2-Mut or novel-miR1 mimics and pmirGLO (**Figure 2G**). Additionally, Western blot analysis indicated that novel-miR1 inhibited the protein expression of TLR2 in PBLCs (**Figures 2H, I**).

### 3.3 Novel-miR1 inhibits the TLR2 signaling pathway and NF-κB activation

**Figure 3:**
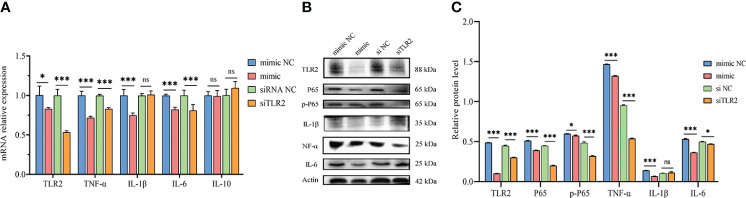
Inhibition of TLR2 signaling pathway and NF-κB activation by novel-miR1. **(A)** qPCR results demonstrate the reduction of *TLR2, TNF-α, IL-1β, and IL-6* mRNA levels by novel-miR1. Each test was performed in triplicate. Fold change was calculated based on qPCR results. **(B)** Western blot assay results indicate the decrease in TLR2, P65, pP65, TNF-α, IL-1β, and IL-6 protein levels by novel-miR1. **(C)** Western blot assay results showing the decrease in protein levels of TLR2, P65, pP65, TNF-α, IL-1β, IL-6 (fold change was calculated by the gray values of the image). ns, p ≥ 0.05; *, p ≤ 0.05; ***, p ≤ 0.001.

TLR2 is a pattern recognition receptor that plays a critical role in initiating innate inflammatory responses and promoting adaptive immune responses (38, 39). To investigate the effect of novel-miR1 targeting TLR2 in rabbit PBLCs, novel-miR1 was overexpressed in rabbit PBLCs via transfection with novel-miR1 mimic. Subsequently, the expression of cytokines related to the TLR2 signaling pathway and NF-κB protein was assessed. The experimental outline is shown in **Figure 3**.

The qPCR results indicated significant downregulation of *TLR2, IL-6, TNF-α*, and *IL-1β* mRNA expression in rabbit PBLCs. As a control, TLR2 siRNA was transfected into rabbit PBLCs, which confirmed that the mRNA expression of *TLR2* was downregulated. Interestingly, the expression of *IL-6* and *TNF-α* was significantly downregulated (**Figure 3A**). Consistent with the qPCR results, Westen blot results showed that the protein expression of TLR2 IL-6, TNF-α, and IL-1β was also downregulated. Additionally, we observed downregulation of P65 and phosphorylated-P65 (pP65) protein levels (**Figures 3B, C**), indicating inhibition of the NF-κB pathway activation due to reduced TLR2 expression. These results collectively suggest that novel-miR1 inhibits the NF-κB pathway signaling by targeting TLR2.

**Figure 4:**
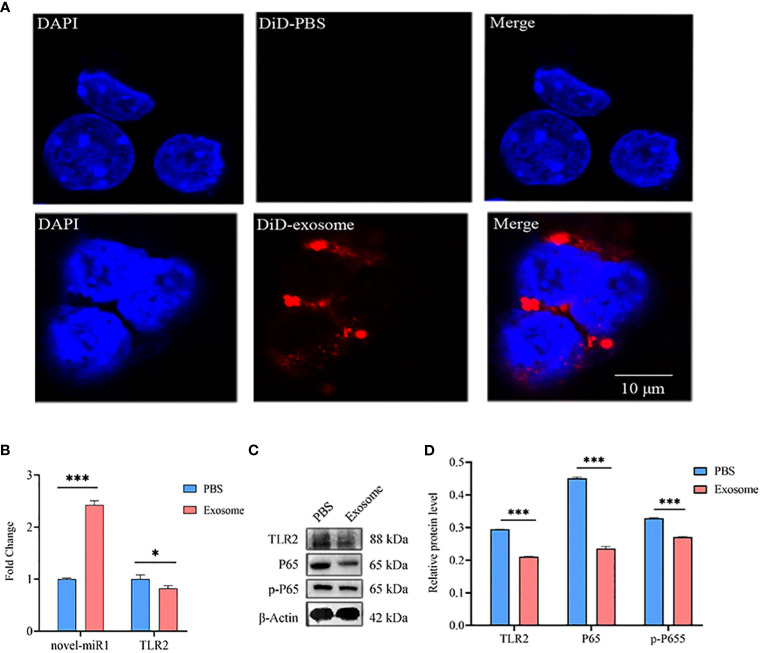
*C. pisiformis* exosomes inhibit the TLR2 signaling pathway and NF-κB activation. **(A)** *C. pisiformis* exosomes can enter rabbit PBLCs. **(B)** qPCR results demonstrate the increased expression of novel-miR1 and decreased *TLR2* mRNA level in rabbit PBLCs after treatment with *C. pisiformis* exosomes. Each test was performed in triplicate; fold change was calculated based on qPCR results. **(C)** Western blot assay results suggest that treatment with *C. pisiformis* exosomes decreased TLR2, P65, and pP65 protein expression levels. **(D)** Western blot assay results indicating the reduction in protein expression levels of TLR2, P65, and pP65 by *C. pisiformis* exosomes (fold change was calculated by the gray values of the image). *, p ≤ 0.05; ***, p ≤ 0.001.

Next, *C. pisiformis* exosomes were marked with DiD dye and cocultured with rabbit PBLCs, which revealed that *C. pisiformis* exosomes can enter rabbit PBLCs (**Figure 4A**). Furthermore, qPCR results suggested that the expression of novel-miR1 and TLR2 was inhibited by *C. pisiformis* exosomes (**Figure 4B**). Similarly, Western blot results showed that *C. pisiformis* exosomes downregulated the expression of TLR2, P65, and pP65 proteins (**Figures 4C, D**).

**Figure 5:**
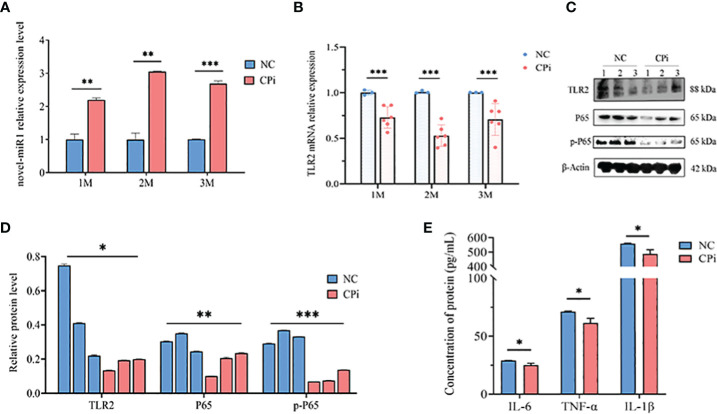
*C. pisiformis* inhibits the TLR2 signaling pathway and NF-κB activation. **(A)** Expression profile of novel-miR1 in rabbit PBLCs over a 3-month period. **(B)** Relative expression TLR2 mRNA in rabbit PBLCs during the initial 3-month period. **(C)** Western blot assay results indicate inhibition of TLR2, P65, and pP65 protein levels in *C. pisiformis*-infected rabbit PBLCs. **(D)** Western blot assay results demonstrating the inhibition of TLR2, P65, and pP65 protein levels in *C. pisiformis*-infected rabbit PBLCs (the fold change was calculated by the gray values of the image). **(E)** ELISA assay results indicate inhibition of IL-6, TNF-α, and IL-1β protein expression levels in *C. pisiformis*-infected rabbit PBLCs. *, p ≤ 0.05; **, p ≤ 0.01; ***, p ≤ 0.001.

Lastly, rabbit PBLCs were isolated from the CPi and NC groups for detection and analysis. The results revealed consistent upregulation of novel-miR1 expression and downregulation of TLR2 mRNA during the initial 3 months (**Figures 5A, B**). Similarly, protein levels of TLR2, P65, pP65, IL-6, TNF-α, and IL-1β were downregulated (**Figures 5C–E**). In summary, these findings strongly support the release of novel-miR1 into the bloodstream through *C. pisiformis* exosomes in infected rabbits and demonstrate that novel-miR1 targets TLR2 to inhibit NF-κB pathway signaling.

## 4 Discussion

In our previous study, we observed a significant upregulation of novel-miR1 in rabbit serum through high-throughput sequencing of small RNA, which was further validated by qPCR. However, the sources of novel-miR1 within rabbit serum have not been well elucidated. In the present study, we conducted absolute quantification qPCR to assess total miRNA levels in samples from rabbit serum (CPi and NC), *C. pisiformis*, and *C. pisiformis* exosomes. The results indicated a significant enrichment of novel-miR1 in *C. pisiformis* exosomes. However, it is important to note that higher copy numbers observed in the NC group samples may be attributed to nonspecific sequence amplification.

To further elucidate the functions of novel-miR1, we employed transfection of novel-miR1 mimics and inhibitors into rabbit PBLCs. Interestingly, we were unable to detect any differences in novel-miR1 or novel-miR1 target gene expression in the groups transfected with novel-miR1 inhibitors (**Supplementary Figures S1, S2**). This finding suggests that novel-miR1 may not naturally exist within rabbit PBLCs and provides supporting evidence that rabbit serum novel-miR1 is released by *C. pisiformis*.

Our data revealed that the expression level of novel-miR1 increased up to 2 months and then decreased by the third month, while TLR2 mRNA expression followed an opposite trend, decreasing until the second month and subsequently increasing. The upregulation of novel-miR1 during the initial stages of infection and the subsequent decrease may be indicative of an ongoing enhancement of immune interactions between *C. pisiformis* and rabbits. After 2 months of infection, the immune response gradually weakens, facilitating continued parasitism by *C. pisiformis*. Additionally, the opposite expression trends observed between novel-miR1 and TLR2 provide evidence that TLR2 is a target gene of novel-miR1.

The findings in the present study highlight the significant immunoregulatory role of novel-miR1 by targeting rabbit PBLCs TLR2 during *C. pisiformis* infection. It has been reported that the miRNAs, as regulators of innate immunity, can regulate components of the TLR signaling pathway, including the TLRs themselves, associated signal proteins, regulatory molecules, transcription factors, and functional cytokines (40). For instance, mouse-derived mmu-miR-92a-2-5 can target TLR2 to inhibit *Schistosoma japonicum*-induced liver fibrosis and downregulate the expression of TLR2-related cytokines such as IL-4, IFN-γ, and TNF-α during *S. japonicum* infection in mice (41).

Further investigations have revealed that novel-miR1 can inhibit the expression of TNF-α-, IL-1β, and IL-6-related cytokines in rabbit PBLCs. Notably, novel-miR1 showed significant upregulation in rabbit PBLCs from 1 to 3 months during *C. pisiformis* infection in rabbits, while the corresponding expression of TLR2 was downregulated. TNF-α, IL-1β, and IL-6 play critical roles in regulating worm immune evasion. Cysteine protease inhibitors (cystatins) present in the ESPs of *Fasciola hepatica* were found to reduce the production of IL-6 and TNF-α in mouse macrophages (42). Similar findings have been reported in *S. japonicum* infections (43). Additionally, it has been reported that mice infected with *Echinococcus multilocularis* and treated with albendazole exhibited a reversal of elevated levels of IL-1β, IL-6, TNF-α, and IFN-γ in the liver (44). Inhibition of these inflammatory cytokines tends to regulate the host immune response toward Th2, and numerous studies have demonstrated the beneficial effects of helminths in inhibiting inflammatory cytokines for the management of auto-inflammatory/immune diseases (45–48).

Based on our study, we have identified that novel-miR1 has significant therapeutic potential for the treatment of allergic, inflammatory, and autoimmune disorders in rabbit. Furthermore, further investigation into the regulatory mechanisms governed by novel-miR1 holds potential for the prevention and treatment of human auto-inflammatory/immune diseases.

## 5 Conclusion

**Figure 6:**
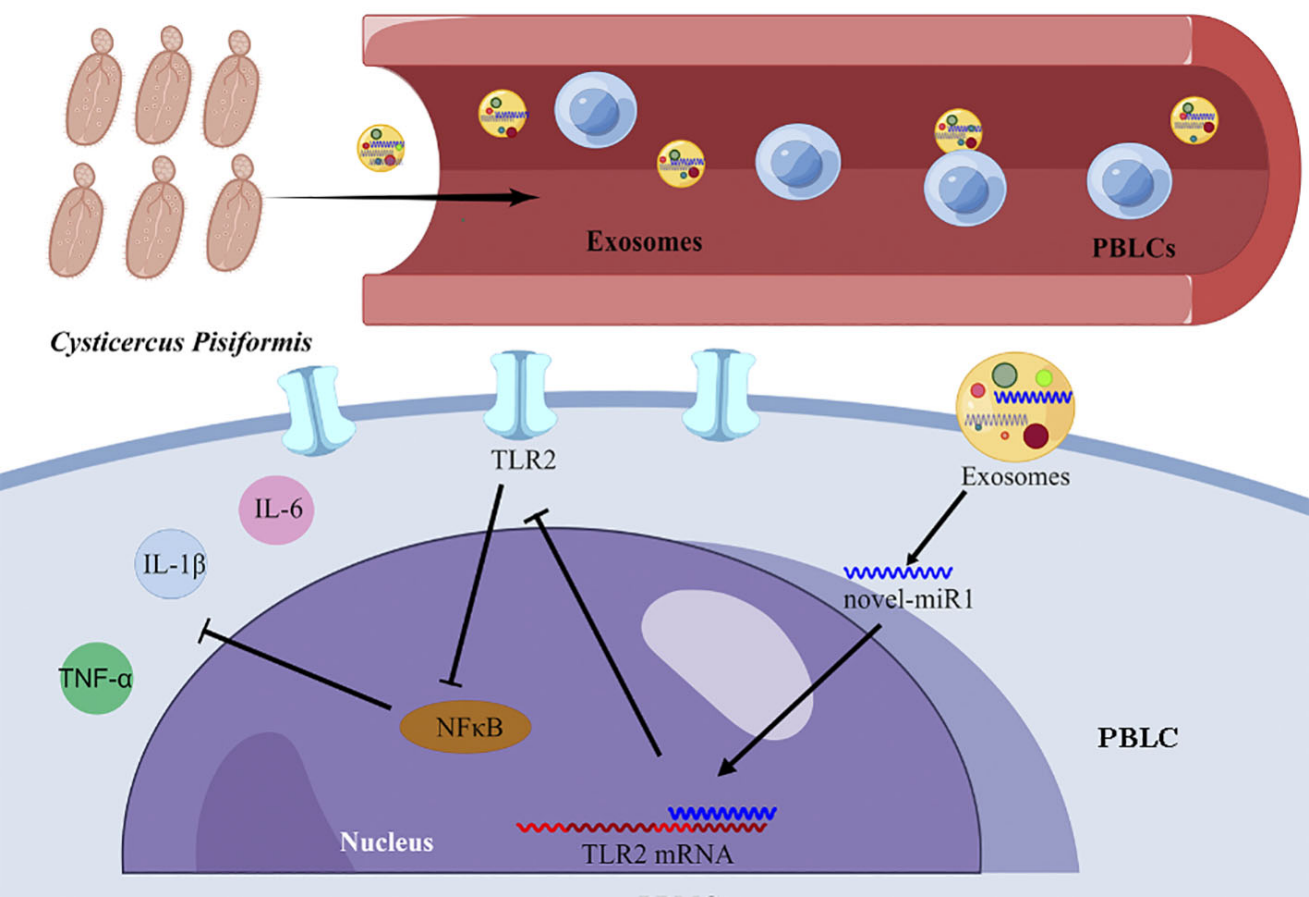
Graphical representation illustrating the mechanism of novel-miR1 targeting TLR2 in PBLCs to regulate the immune response in rabbits. The figure was created using Figdraw.

The results from the present study strongly indicate that *C. pisiformis-*derived exosomes serve as a means for the release of novel-miR1 into rabbit serum during parasite infection. Moreover, our results demonstrate that novel-miR1 exerts its regulatory effects by inhibiting the expression of TLR2, thereby modulating the production of pro-inflammatory factors via the NF-κB pathway. These observations support the notion that novel-miR1 plays a crucial role in facilitating parasite survival and establishment within the host (**Figure 6**).

## Data availability statement

The original contributions presented in the study are included in the article/**Supplementary Material**. Further inquiries can be directed to the corresponding author.

## Ethics statement

The animal study was reviewed and approved by Animal Ethics Committee of Lanzhou Veterinary Research Institute (SYXK 2020–0010).

## Author contributions

GC and XNL conceived and designed the study, analyzed the data, drafted and critically revised the manuscript. GC, GP, LW performed the experiments. YL, LW, TL, HL, GP, SZ, XW, and XLL helped in the implementation of the study and performed and interpreted the computational analysis. All authors have read and approved the final manuscript.

## Supplementary material

Supplementary Figure 1 Relative expression of novel-miR1 in rabbit PBLCs treated with novel-miR1 inhibitors.

Supplementary Figure 2 qPCR results showing the relative expression of TLR2, TNF-α, IL-1β, IL-6, and IL-10 mRNA in rabbit PBLCs treated with novel-miR1 inhibitors.

Supplementary Table 1 Quantification of *C. pisiformis in* rabbits from CPi and NC groups.

Supplementary Table 2 Prediction of target genes for novel-miR1 using Targetscan, miRNADA and PITA.

Supplementary Table 3 Functional annotation clustering analysis of novel-miR1 target genes using GO.

Supplementary Table 4 KEGG pathway analysis of novel-miR1 target genes.
