# Supplementary figures and images for: Cysticercus pisiformis-derived novel-miR1 targets TLR2 to inhibit the immune response in rabbits
Source: Front Immunol. 2023 Jul 25;14:1201455. doi: 10.3389/fimmu.2023.1201455 (PMC10408446; doi:10.3389/fimmu.2023.1201455)

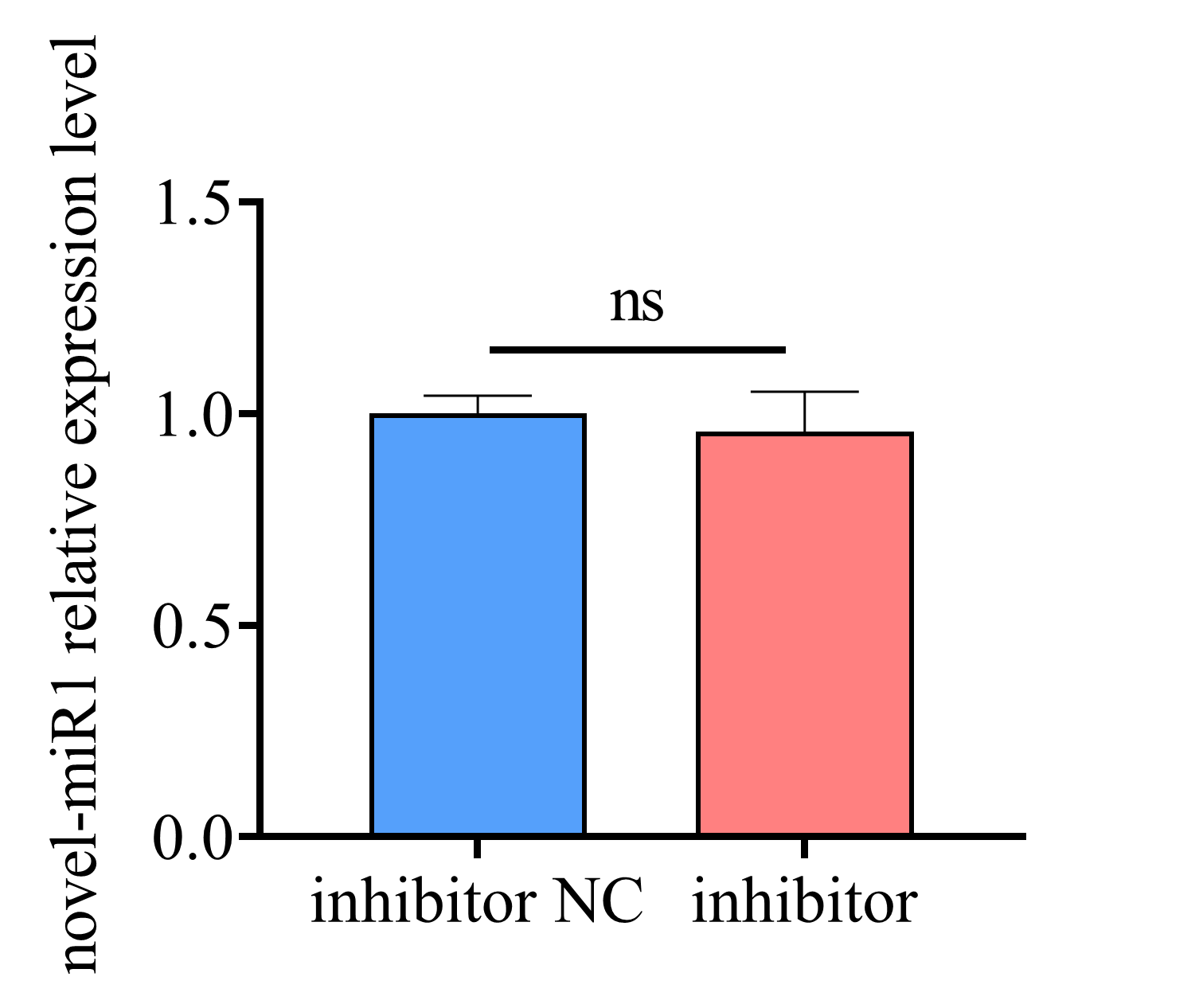

Supplement: Supplementary Figure 1 — Relative expression of novel-miR1 in rabbit PBLCs treated with novel-miR1 inhibitors. [file Image_1.tif]

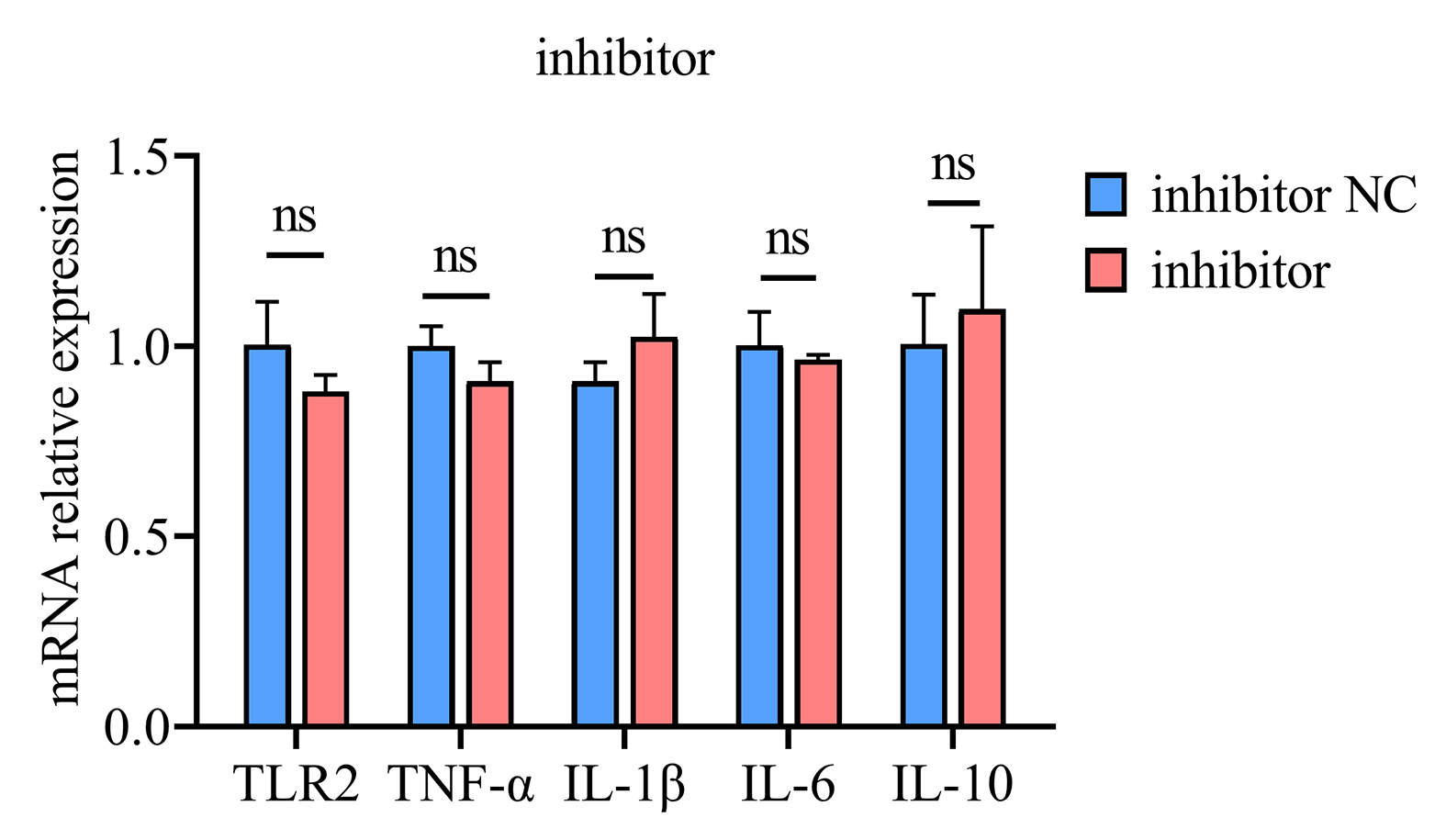

Supplement: Supplementary Figure 2 — qPCR results showing the relative expression of TLR2, TNF-α, IL-1β, IL-6, and IL-10 mRNA in rabbit PBLCs treated with novel-miR1 inhibitors. [file Image_2.tif]
